# Artificial Graphite-Based Silicon Composite Anodes for Lithium-Ion Batteries

**DOI:** 10.3390/nano14231953

**Published:** 2024-12-05

**Authors:** Sae Min Park, Tejaswi Tanaji Salunkhe, Ji Hyeon Yoo, Il Ho Kim, Il Tae Kim

**Affiliations:** 1 Department of Chemical, Biological and Battery Engineering, Gachon University, Seongnam-si 13120, Gyeonggi-do, Republic of Korea; ssmmp0101@daum.net (S.M.P.); tejaswisalunkhe235@gmail.com (T.T.S.); gina010219@naver.com (J.H.Y.); 2 R&D Center, Black Materials Co., Ltd., Hwaseong-si 18255, Gyeonggi-do, Republic of Korea; gsystem@hanmail.net

**Keywords:** Li-ion batteries, anode, artificial carbon, high-energy ball milling

## Abstract

To develop an advanced anode for lithium-ion batteries, the electrochemical performance of a novel material comprising a porous artificial carbon (PAC)–Si composite was investigated. To increase the pore size and surface area of the composite, ammonium bicarbonate (ABC) was introduced during high-energy ball-milling, ensuring a uniform distribution of silicon within the PAC matrix. The physical and structural properties of the developed material were evaluated using several advanced techniques, including X-ray diffraction (XRD), transmission electron microscopy (TEM), and galvanostatic intermittent titration (GITT). Artificial graphite contains several macropores that can accommodate volume hysteresis and provide effective sites for anchoring Si nanoparticles, enabling efficient electrochemical reactions. GITT analysis revealed that the PAC-Si-CB-ABC composite exhibited superior lithium-ion diffusion compared to conventional graphite. The developed PAC(55%)-Si(45%)-CB-ABC electrode with PAA as the binder demonstrated a reversible capacity of 850 mAh g^−1^ at 100 mA g^−1^ and a high-rate capability of 600 mAh g^−1^ at 2000 mA g^−1^. A full cell employing the NCM622 cathode exhibited reversible cyclability of 128.9 mAh g^−1^ with a reasonable energy density of 323.3 Wh kg^−1^. These findings suggest that the developed composite is a useful anode system for advanced lithium-ion batteries.

## 1. Introduction

The demand for high-capacity and efficient battery anode materials has significantly increased in recent years, driven by the rapid growth of portable electronic devices, electric vehicles, and renewable energy storage systems. Among various available carbon materials, artificial graphite is one of the most promising candidates for energy storage applications, particularly for use in lithium-ion batteries (LIBs), because of its relatively stable structure, good electrical conductivity, and moderate cost [1,2]. However, despite these advantages, the theoretical capacity of artificial graphite (372 mAh g^−1^) is relatively low compared to that of other potential anode materials, making it less suitable for next-generation, high-capacity applications such as electric vehicles and high-performance portable electronics [3]. Moreover, artificial graphite often suffers from a significant initial irreversible capacity loss, primarily because of its complex and challenging surface structure, which leads to the formation of a thick solid electrolyte interphase (SEI) layer that consumes active lithium and diminishes the overall battery efficiency [4,5].

To overcome these limitations, considerable research has been directed toward the development of silicon-based anode materials, including silicon monoxide (SiO), silicon alloys, and silicon-carbon nanocomposite materials [6]. Si, with a theoretical capacity of approximately 4200 mAh g^−1^, offers a substantial improvement over graphite, making it an attractive alternative for high-energy-density applications [7]. However, silicon anodes face challenges such as significant volume expansion (up to ~300%) during lithiation, which can lead to mechanical degradation, loss of electrical contact, and rapid capacity fading during cycling [8]. Recent advancements in materials science have focused on mitigating these issues by creating nanoscale micropores and incorporating Si into composite structures with carbon materials to improve the energy density and charge–discharge rate capabilities of the anode [9,10].

With this scenario, in this study, porous artificial graphite (PAC) derived from anthracite, provided by Black Materials Co., Ltd., is processed using a high-energy ball milling technique to reduce the particle size to below 500 μm. The fine particles are then separated from the floating matter using a Denver-type screening method to ensure a uniform particle size distribution. The resulting material is subjected to high-temperature treatment above 2850 °C for 3 h, which facilitates the formation of a PAC structure with enhanced mechanical and electrochemical properties [11,12].

To further increase the capacity and conductivity, silicon nanoparticles are incorporated into PAC, and acetylene black (CB) is introduced as a conductive additive to enhance the electrical pathways within the composite material [13,14]. Moreover, ammonium bicarbonate (ABC) is introduced as a foaming agent to increase the pore size and surface area of the synthesized material. Ammonium bicarbonate, widely used across various industries, is known to decompose at low temperatures (around 36 °C), breaking down into carbon dioxide, ammonia, and water vapor. The gases generated during decomposition create bubbles within the material, leading to volume expansion and the formation of a highly porous structure [15,16]. This increase in porosity is critical for improving the ion mobility within the electrode material, which, in turn, enhances the surface area and overall electrochemical performance of the electrode. Ammonium bicarbonate also offers practical advantages owing to its low cost and easy availability, making it an economical option for the large-scale production of high-performance battery materials. Notably, however, the gases generated during ABC decomposition must be carefully removed through subsequent heat treatment to ensure the structural integrity and electrochemical stability of the final product.

By integrating these advanced synthesis techniques, this study aims to develop a high-performance anode material that combines the benefits of the high capacity of silicon with the structural and conductive advantages of PAC. The resulting PAC-Si-CB-ABC composite is expected to offer substantial improvements in energy density, cycle life, and rate capability, making it a promising candidate for anodes of next-generation lithium-ion batteries that can meet the demands of modern energy storage applications.

## 2. Experimental Section

### 2.1. Synthesis of PAC-Si-CB-ABC Composite

**Figure 1:**
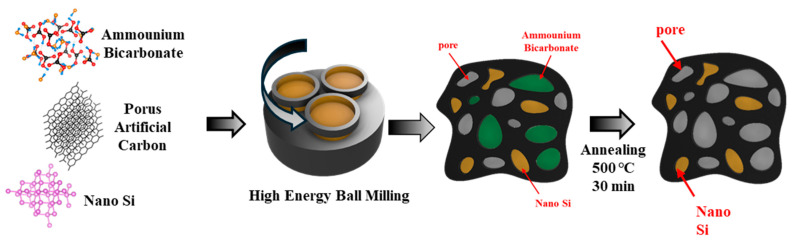
Schematic of high-energy ball milling process for synthesis of PAC-Si-CB-ABC composite.

The PAC-Si-CB-ABC active material was synthesized using Si powder (crystalline, 100 nm, 99%, Plasma, Alfa, China), ammonium bicarbonate (NH_4_HCO_3_, 99.0%, Aldrich, Darmstadt, Germany), acetylene black (200 mesh, 99.8%, Aldrich, Shanxi, China), and PAC. The proportions used were 73.8% ABC, 7.7% acetylene black, and a mixture of PAC and Si in varying ratios totaling 18.4%. Specifically, the ratios of PAC to Si were 25:75, 40:60, 55:45, 70:30, and 85:15, which resulted in five samples. Each of the materials was mixed together and placed in a zirconia crucible (80 cm^3^) with ZrO_2_ balls (diameters of 0.5 inches and 0.25 inches). High-energy ball milling (HEBM) was performed under an argon atmosphere for 10 h at 300 rpm using a Pulverisette 5 Planetary Mill (Fritsch GmbH, Idar-Oberstein, Germany). Following milling, the materials were heated to 500 °C for 30 min under argon atmosphere in a furnace to remove the ABC component from the mixture. The overall preparation is illustrated in Figure 1. The product was pulverized in a grinder to ensure uniformity of the active material. To fabricate the anode, the prepared powder was mixed with a binder (PVDF, Aldrich, 12 wt% in NMP or PAA, Aldrich, 99.99% in ethanol) and a conductive agent (Super P) in a mass ratio of 70:15:15. The slurry was cast onto a copper foil substrate and dried in a vacuum oven at 70 °C. After drying for approximately 15 min, the electrode was removed from the oven and roll-pressed at 70 °C with a 0.05 mm gap. The electrode was then placed in a vacuum oven and dried for approximately 24 h. The dried electrode was punched into discs with a diameter of 12 mm; the loading amount was 0.80–1.10 mg cm^−2^. The electrodes were then assembled into CR2032 coin cells in a glove box using a polyethylene separator and a lithium foil as the counter electrode. LiPF_6_ (1 M in ethylene carbonate (EC):diethyl carbonate (DEC) = 1:1, volume ratio) was used as the electrolyte (~120 mL) with and without an additive (5 vol.% FEC).

### 2.2. Materials Characterization

The synthesized materials were characterized by various analytical techniques. X-ray diffraction (XRD; instrument facility at the Smart Materials Research Center for IoT at Gachon University) data were acquired using a D/max 2200 (Rigaku, Japan) instrument, at a scan rate of 3 °C min^−1^, over the temperature range of 10–890 °C. To analyze the microstructure and composition of the samples, transmission electron microscopy (TEM) with energy-dispersive X-ray spectroscopy (EDX) and high-resolution TEM (HRTEM) data were obtained using JEOL JEM 2100 (JEOL, Japan) instrument. The surface area and porosity of the materials were determined by the Brunauer–Emmett–Teller (BET) method at 150 °C using a ASAP 2020 analyzer (Micromeritics, United State).

### 2.3. Electrochemical Measurements

All tests were conducted using a WNCS3000 battery cycler (WonAtech, Seoul, Republic of Korea) at 25 °C with a current density of 100 mA g^−1^, within the voltage range of 0.01–2.0 V (Li vs. Li^+^). Galvanostatic intermittent titration technique (GITT) and cyclic voltammetry (CV) measurements were performed using a ZIVE system (WonAtech, Seoul, Republic of Korea).

## 3. Results and Discussion

**Figure 2:**
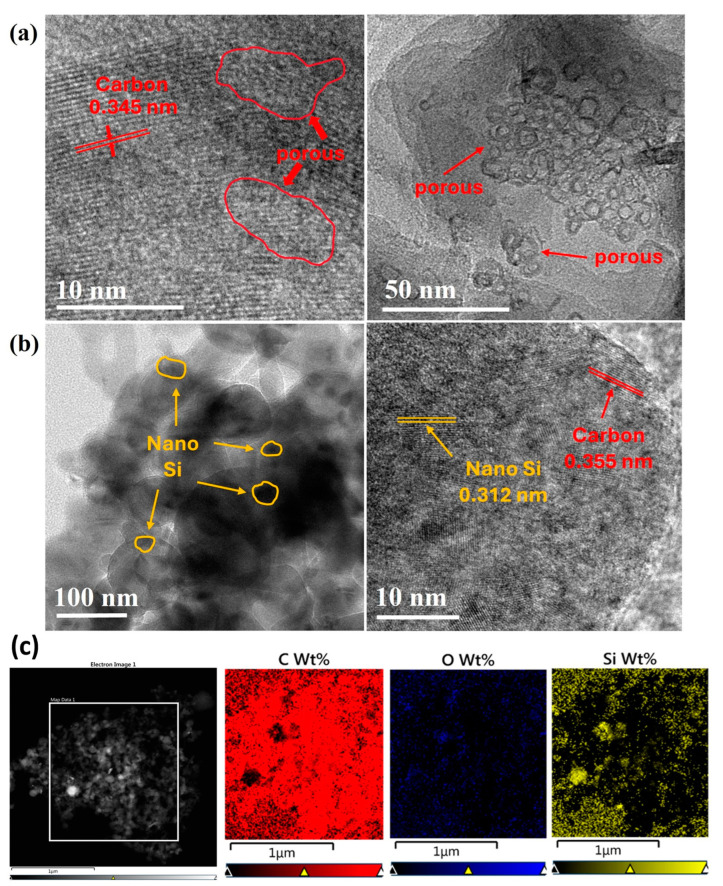
(**a**) TEM image of PAC, (**b**) PAC(55%)-Si(45%)-CB-ABC, and (**c**) EDS mapping of PAC(55%)-Si(45%)-CB-ABC.

The microstructure, other structural characteristics, and elemental distributions of the synthesized samples were examined using TEM, EDX, and HRTEM. The TEM images confirmed that the artificial graphite had a porous structure. Pores of various sizes [17] were present within the sample (Figure 2a). When observing Figure 2a, the distribution of pores is not uniform. During the annealing process, the ABC embedded in the PAC was removed, leaving behind these porous features. As seen in Figure 2a, the pores are clustered in some regions, and these clusters are observed sporadically throughout the sample. The pores increased the pathways for lithium ions and expanded the surface area between the electrode material and the electrolyte, which are beneficial features for enhancing the battery performance.

The post-milling TEM images revealed that the Si particles were evenly distributed within the PAC (Figure 2b), demonstrating that the milling process effectively incorporated Si into the porous structure. The detection of oxygen in the EDS map suggests that silicon was oxidized. Oxidized silicon can contribute to stabilizing the solid electrolyte interphase (SEI) layer, facilitating smoother lithium-ion movement, and potentially enhancing the cycle life of the battery [18,19].

Moreover, the oxidation of Si could help mitigate volume changes, thereby increasing the mechanical stability of the electrode, and reducing direct reactions with the electrolyte, preventing the degradation of silicon and improving the durability of the electrode. Thus, silicon oxide could enhance the overall stability of batteries by effectively reducing the risk of thermal runaway and side reactions, thereby enabling reliable use over long periods [20]. Silicon was oxidized owing to the inclusion of ABC, which provides oxygen during the milling process.

**Figure 3:**
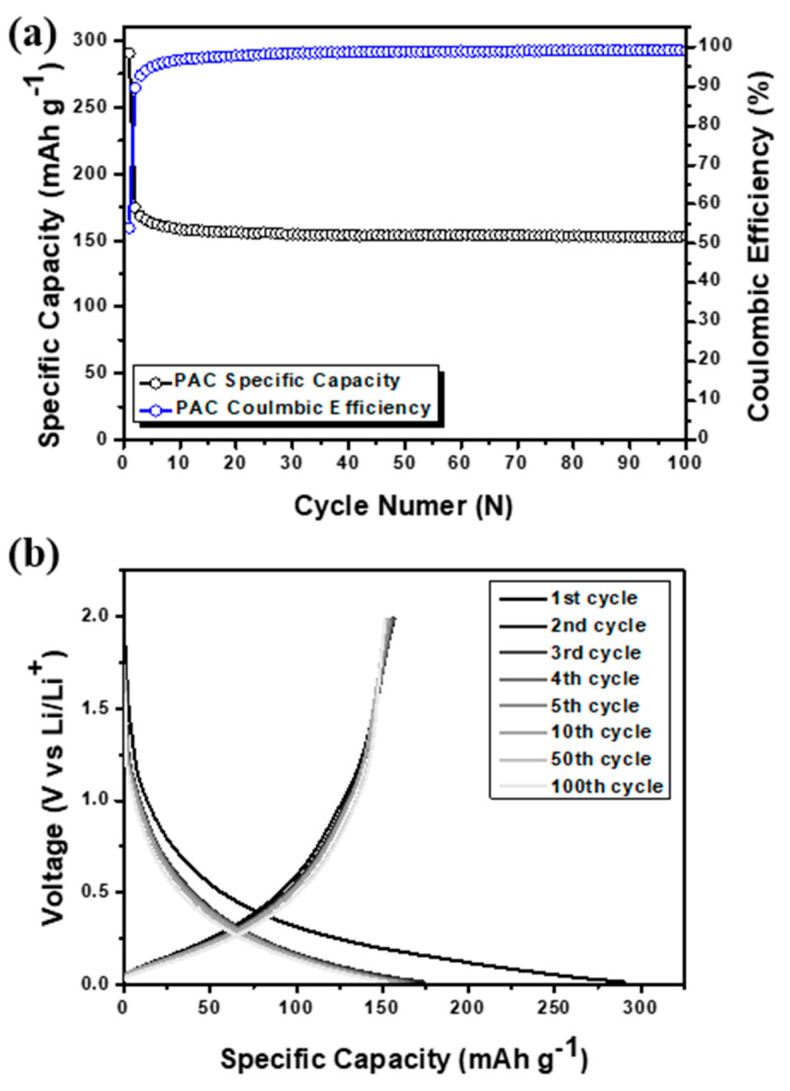
(**a**) Cycle performance with coulombic efficiency and (**b**) voltage profile of pure PAC.

Despite its high stability, the use of PAC as an anode material is limited owing to its low capacity. Specifically, PAC exhibited a reversible capacity of only 150 mAh g^−1^, which is lower than that of the theoretical capacity of graphite (372 mAh g^−1^) [21]. This considerable capacity deficit, as shown in Figure 3, makes PAC less suitable for commercial applications and high-capacity electronic devices, despite its excellent stability over extended periods and repeated charge–discharge cycles and its low cost. In addition, the anode material consisting of Si-CB-ABC without PAC was prepared, which revealed a dramatic capacity fading, revealing a very low capacity of approximately 50 mAh g^−1^ as shown in Figure S1.

To overcome this limitation, the anode capacity can be enhanced by incorporating silicon into the PAC matrix during the milling process, thus forming a carbon-silicon composite. Si was selected because of its high theoretical capacity, which can potentially mitigate the structural deficiencies of PAC. Concurrently, the artificial graphite component is expected to mitigate volumetric expansion issues typically associated with Si, thereby enhancing the overall structural integrity of the anode material [22]. ABC was added to the composites to improve the charge and discharge efficiencies. By volatilizing ABC at high temperatures, the pore size of the material was significantly enhanced, which, in turn, widened the pathways for lithium ions. This modification is expected to facilitate more efficient ion movement, thereby enhancing the electrochemical performance of the developed electrodes [23].

**Figure 4:**
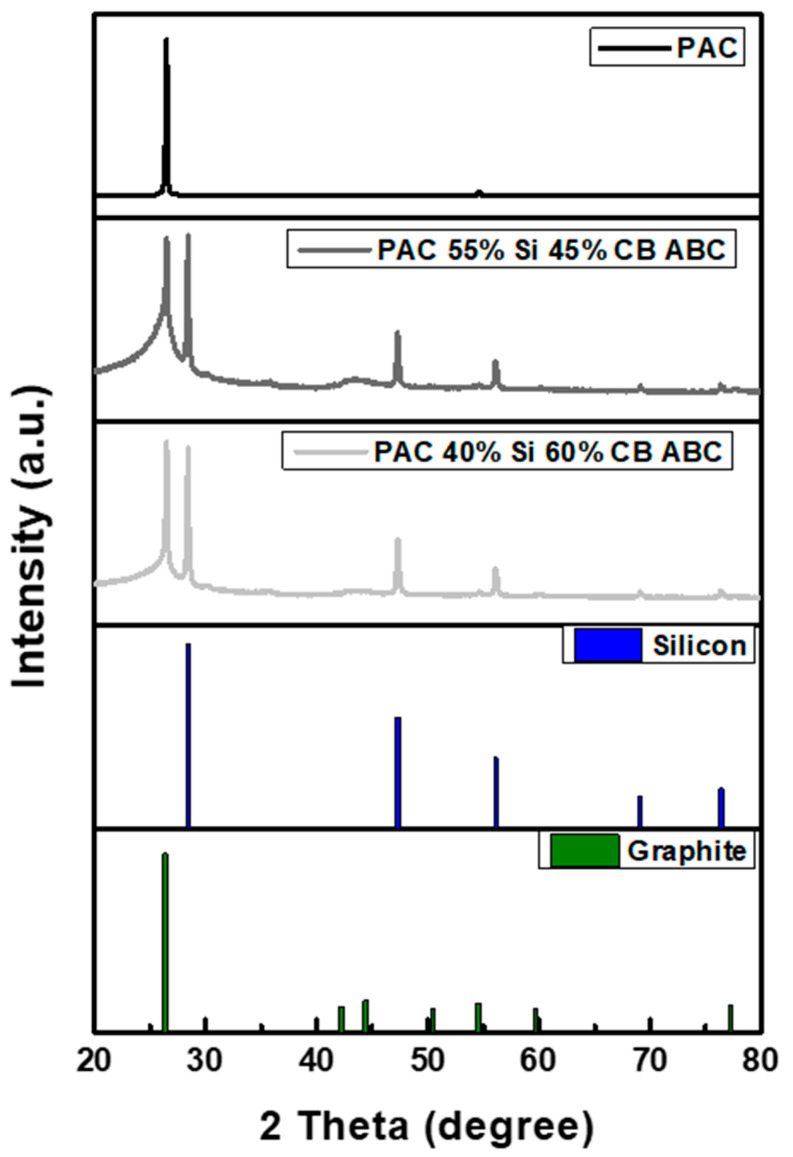
XRD patterns of PAC, PAC(55%)-Si(45%)-CB-ABC, and PAC(40%)-Si(60%)-CB-ABC.

The phase structure of the PAC-Si-CB-ABC composite was analyzed using powder X-ray diffraction (XRD). As illustrated in Figure 4, the physicochemical compositions of the artificial graphite and other samples were examined. The prominent diffraction peaks at 26.381° and 54.542° correspond to the (002) and (004) planes, respectively, which are the characteristic peaks of artificial graphite [24]. Additionally, the diffraction peaks at 28.442°, 47.302°, 56.121°, 69.13°, and 76.377° correspond to the (111), (220), (311), (400), and (331) planes, respectively, indicating the presence of nanosized silicon particles [25]. The observed peak broadening suggests a reduction in the crystallinity and a more amorphous structure, suggesting that the particles were finely milled and that the crystal structure was modified during the milling process. XRD analysis confirmed that the Si particles existed with the artificial graphite matrix.

**Figure 5:**
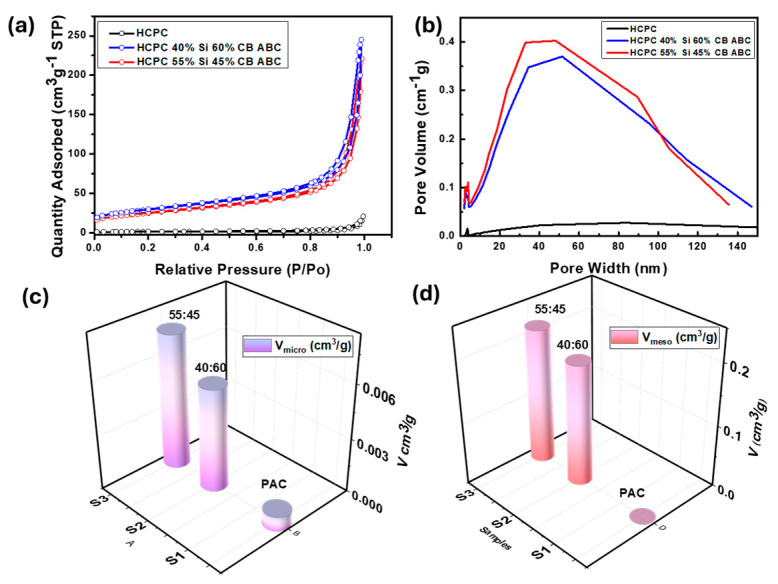
Specific surface area of PAC, PAC-Si, PAC(55%)-Si(45%)-CB-ABC, and PAC(40%)-Si(60%)-CB-ABC. (**a**) Adsorption/desorption isotherm plot of BJH, (**b**) pore size distribution, (**c**) micropore and (**d**) mesopore volume comparison.

The surface area of the synthesized active material was measured at 150 °C using the Brunauer–Emmett–Teller (BET) method. The pore volume of the artificial graphite was 0.023439 cm^3^ g^−1^, whereas the pore volume of the synthesized active material increased significantly, averaging 0.276838 cm^3^ g^−1^, which is more than ten times greater [26]. This substantial increase in the pore volume indicates that the pores expanded significantly owing to the removal of ABC. During the annealing process, ABC generally evaporates and generates gases, including NH_3_, CO_2_, and H_2_O [15,27]. This gas generation can develop porous sites, leading to a significant increase in the pore volume. The increase in the pore volume indicates that the pore structure was well-formed during the synthesis, particularly owing to the role of ABC, which expanded the existing volume structure and affected the electrochemical properties of the composite. The BET surface area and pore volume data clearly indicate the evolution of porosity in the samples. The pristine carbon (PAC) exhibited a low BET surface area of 5.122 m^2^ g^−1^, with negligible micropore (0.0008 cm^3^ g^−1^) and mesopore volumes. In contrast, the hybrid samples, PAC(40%)_Si(60%)_ABC_CB and PAC(55%)_Si(45%)_ABC_CB, showed significantly higher BET surface areas of 88.167 m^2^ g^−1^ and 104.293 m^2^ g^−1^, respectively [5]. The micropore volumes increased to 0.0057 cm^3^ g^−1^ and 0.0075 cm^3^ g^−1^, while the mesopore volumes increased to 0.194 cm^3^ g^−1^ and 0.217 cm^3^ g^−1^, respectively (Figure 5c,d). These results confirm the generation of a hierarchical porous structure, including both micropores and mesopores, upon the addition of silicon, ABC (acting as a bubble template), and subsequent processing. Ball milling ensures homogeneous mixing, enhancing pore uniformity. The decomposition or evaporation of ABC during annealing creates voids, resulting in the observed microporosity. The higher silicon content may hinder the effective dispersion of ABC during ball milling, leading to less efficient pore formation, as silicon clusters can block bubble template access. The higher silicon fraction increases the density of the composite, which could partially collapse or block existing pores during annealing. Consequently, mesopores and micropores create more pathways for lithium ions to diffuse into the active material, improving the charging and discharging speeds and capacity of the battery. It is noted that PAC has achieved significantly increased porosity compared to its previous state. This enhanced porosity facilitates lithium-ion mobility, thereby improving performance. Additionally, the porous structure contributes to higher capacity and enhanced chemical reaction efficiency in the battery. However, the lack of uniform pore distribution might explain the observed decline in cycle performance (discussed later).

**Figure 6:**
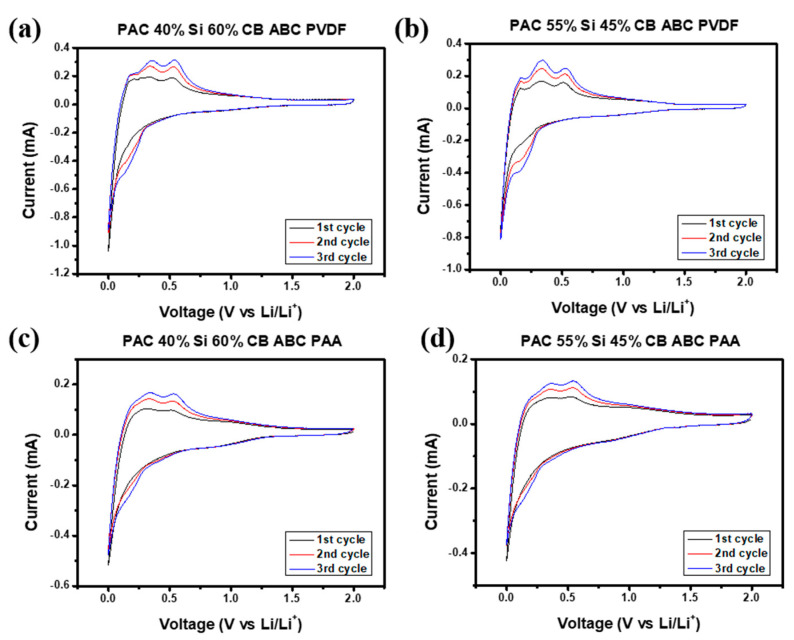
Cyclic voltammograms of (**a**,**b**) PAC(55%)-Si(45%)-CB-ABC and PAC(40%)-Si(60%)-CB-ABC electrodes with PVDF binder, and (**c**,**d**) PAC(55%)-Si(45%)-CB-ABC and PAC(40%)-Si(60%)-CB-ABC electrodes with PAA binder.

To determine the suitability for use in cells, the PAC-Si-CB-ABC electrodes with two different binders, PVDF and PAA, were examined using cyclic voltammetry (CV). During lithiation, lithium ions are intercalated into the graphite layers, forming lithium–graphite compounds. This reaction typically results in a peak at approximately 0.01 V. The peak in Figure 6 confirms the insertion of lithium ions into the graphite structure. During delithiation, lithium ions exit the graphite layers, accompanied by the reverse reaction, which is evidenced by a peak at ca. 0.2 V [28].

The redox reactions of Si can also be analyzed using CV. Silicon undergoes alloying reactions with lithium ions to form various lithium–silicon alloys. This lithiation process begins at approximately 0.2 V, and depending on the structure and composition of silicon, multiple peaks can be observed across various voltage ranges [29]. Conversely, during delithiation, peaks are observed at ca. 0.48 V, as confirmed by Figure 6.

The peaks in the voltage range of 0.3–0.8 V indicate the formation of a solid electrolyte interphase (SEI) layer. The SEI layer is formed by reactions between the electrode material and the electrolyte and plays a critical role in enhancing battery stability. This layer is crucial for preventing additional electrolyte decomposition [4].

The CV profile of the electrode with PVDF as the binder showed more prominent peaks during the initial cycles than that of the congener with PAA. This may indicate stronger electrochemical reactions and a high initial capacity in the former; however, the overall performance was not stable (discussed later). Based on the aforementioned information, the electrochemical reactions of PAC-Si-CB-ABC during charging and discharging are as follows:

[Charge]

$$C+{Li}^{+}+e^{-}\to LiC_{6}\;Si+4.4{Li}^{+}+4.4e^{-}\to {Li}_{4.4}Si$$

[Discharge]

$$LiC_{6}\to C+{Li}^{+}+e^{-}\;{Li}_{4.4}Si\to Si+4.4{Li}^{+}+4.4e^{-}$$

**Figure 7:**
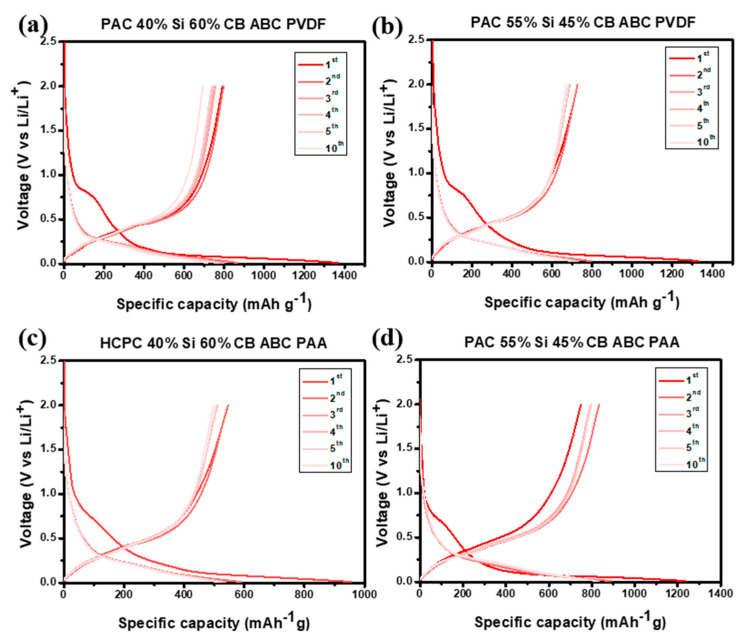
Voltage profiles of (**a**) PAC(40%)-Si(60%)-CB-ABC with PVDF binder, (**b**) PAC(55%)-Si(45%)-CB-ABC with PVDF binder, (**c**) PAC(40%)-Si(60%)-CB-ABC with PAA binder, and (**d**) PAC(55%)-Si(45%)-CB-ABC with PAA binder at a constant current of 100 mA g^−1^.

The voltage profiles of the prepared PAC-Si-CB-ABC electrodes are shown in Figure 7. This measurement was performed in the range of 0.01–2.0 V at a scan rate of 100 mA g^−1^. The initial coulombic efficiency (ICE) was consistently approximately 50–60% (Figure 7), indicating notable losses during the initial charging of the electrode material. These losses are likely due to side reactions including the formation of SEI layer during the insertion of Li ions into the electrode material [30]. The reaction voltages of carbon and silicon matched those obtained in the CV measurements (see Figure 6). For all the electrodes with different binders, the electrochemical reactions were identical; however, the properties of the reversible electrochemical reactions were different, as discussed hereinafter. Despite the low initial coulombic efficiency, subsequent cycles showed high reversible coulombic efficiencies, suggesting a reversible charge–discharge process.

**Figure 8:**
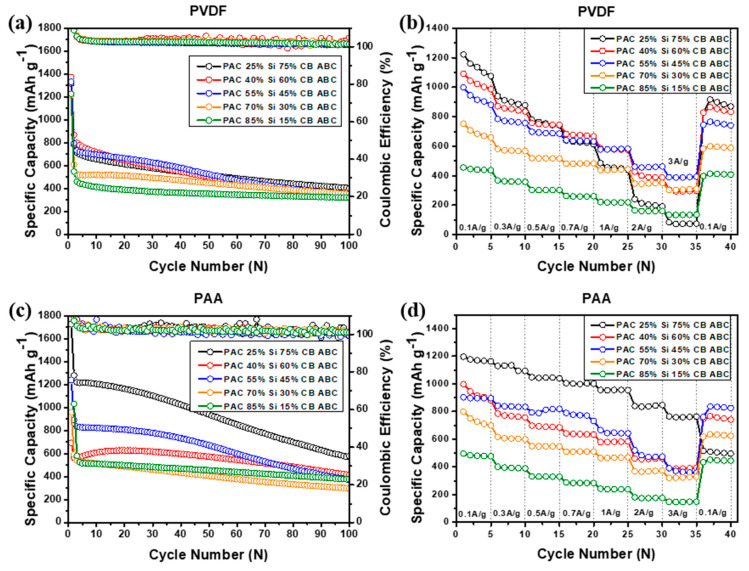
Cyclic performance, coulombic efficiency at 100 mA g^−1^ and rate capability of (**a**,**b**) PAC-Si-CB-ABC electrodes with PVDF binder and (**c**,**d**) PAC-Si-CB-ABC electrodes with PAA binder.

To evaluate the cycle performance of the PAC-Si-CB-ABC electrodes, charge–discharge cycle tests were conducted for over 100 cycles at a current density of 100 mA g^−1^ (Figure 8). The analysis focused on the overall capacity, capacity retention, and efficiency of the different binders. In the case of PAC-Si-CB-ABC with the PVDF binder, the electrodes (for instance, PAC 40% and Si 60%) with a high Si content exhibited higher capacity than those (for instance, PAC 85% and Si 15%) with a low Si content. However, the cyclability tests revealed a gradual capacity decay (Figure 8a). The capacity of the PAC-Si-CB-ABC electrodes with the PAA binder showed a similar tendency, wherein a higher Si content resulted in higher capacity values. The electrodes with the PAA binder demonstrated higher capacity compared to those with the PVDF binder. This difference may be due to the tight binding between Si and PAA, in addition to the conductive medium (CB), as well as the porous sites resulting from ABC evaporation. This was particularly evident in the samples with higher silicon contents, where those with the PAA binder demonstrated moderately improved performance [31]. These synergistic effects generated high reversible capacities during cycling (Figure 8c). PAC(25%)–Si(75%)-CB-ABC with PAA afforded the highest capacity over 100 cycles, albeit with gradual capacity fading. The PAC(55%)-Si(45%)-CB-ABC electrode with PAA showed moderate/more stable cycling performance. The PAC(55%)-Si(45%)-CB-ABC electrode with PAA achieved a capacity of 800 mAh g^−1^, maintaining approximately 80% of the initial capacity after 100 cycles. It is noted that when looking into the CE values, some fluctuations of CE values occurred, illustrating unstable electrochemical reactions. Although the development of large porous sites in the composite electrodes led to a high capacity and reasonable cyclic performance, further tuning of the structure of the composite electrodes is required [31].

The rate capabilities of PAC-Si-CB-ABC with different binders were compared at current densities of 0.1, 0.3, 0.5, and 0.7. 1.0, 2.0, and 3.0 A g^−1^. The electrodes employing the PAA binder exhibited higher stability and reversible capacity than those with the PVDF binder (Figure 8). The best-performing samples were those with Si/C ratios of 40:60 and 55:45. For instance, the PAC(55%)-Si(45%)-CB-ABC electrode with PAA as the binder afforded capacities of 900, 850, 800, 750, 700, 500, and 450 mA g^−1^, respectively, at respective current rates of 0.1, 0.3, 0.5, 0.7, 1.0, 2.0, and 3.0 A g^−1^, where the capacity retention at 3.0 A g^−1^ was ca. 50%. The enhanced performance of the electrodes with the PAA binder is attributed to the strong binding properties of PAA, which enhance the mechanical stability of the electrode and reduce structural deformation during the charge–discharge cycles. The superior mechanical properties of PAA help mitigate volume changes in Si-based anode materials, thereby maintaining the electrode integrity during cycling [32]. As discussed, among the prepared electrodes, PAC(40%)-Si(60%)-CB-ABC and PAC(55%)-Si(45%)-CB-ABC with the PAA binder afforded a greater improvement in the performance. The electrochemical analyses discussed hereafter were conducted with the PAC(40%)-Si(60%)-CB-ABC and PAC(55%)-Si(45%)-CB-ABC electrodes [33].

GITT is an electrochemical method used to analyze and calculate the ion diffusion coefficient in battery electrodes. This technique involves applying a constant current to the battery and periodically interrupting the current to allow the system to reach equilibrium. Information on the ion diffusion process can be obtained by measuring the voltage changes during these interruptions. GITT measurements enable the determination of the ion diffusion coefficients and provide information on the electrochemical characteristics of electrode materials. This enables prediction of the charge–discharge rates, efficiency, and battery lifespan. GITT also enables accurate measurement of the ion diffusion coefficients without disassembling the battery [34]. Weppner and Huggins stated that the diffusion coefficient of Li ions is based on Fick’s second law.

D=4π(iVmFSZA)2[dEdδdEdt]2for t≪L2DD=4πτ(nmVmS)2(∆Es∆Et)2

**Figure 9:**
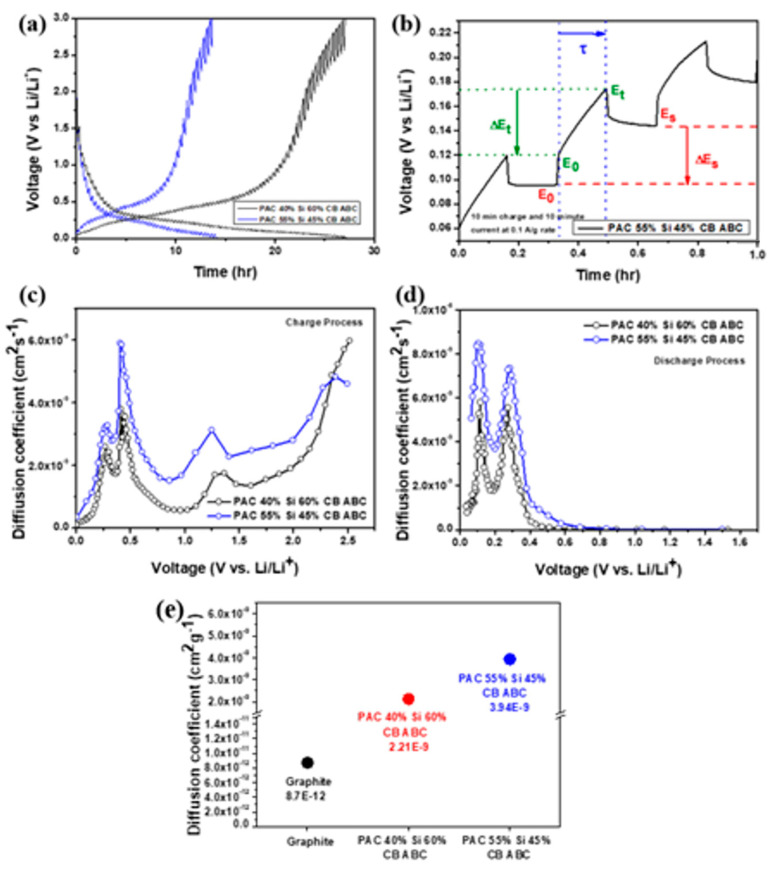
(**a**) GITT curve of PAC(40%)-Si(60%)-CB-ABC and PAC(55%)-Si(45%)-CB-ABC electrodes with PAA binder, (**b**) potential and time differences, calculated diffusion coefficient during (**c**) charge and (**d**) discharge processes, and (**e**) comparison of diffusion coefficients.

The lithium-ion diffusion coefficients of the PAC(40%)-Si(60%)-CB-ABC and PAC(55%)-Si(45%)-CB-ABC electrodes were, respectively, 2.21 × 10^−9^ and 3.94 × 10^−9^ (Figure 9), which are significantly higher than the typical diffusion coefficient of graphite (8.7 × 10^−12^) [35].

Thus, the anodes can facilitate faster ion movement, thereby enhancing the high-rate performance. The increased surface area and porosity due to the addition of ABC to the composites, along with the uniform dispersion of silicon during the milling process, contributed to these results. The results highlight the potential of the material as a superior anode for improving the battery efficiency, capacity, and cycle life.

**Figure 10:**
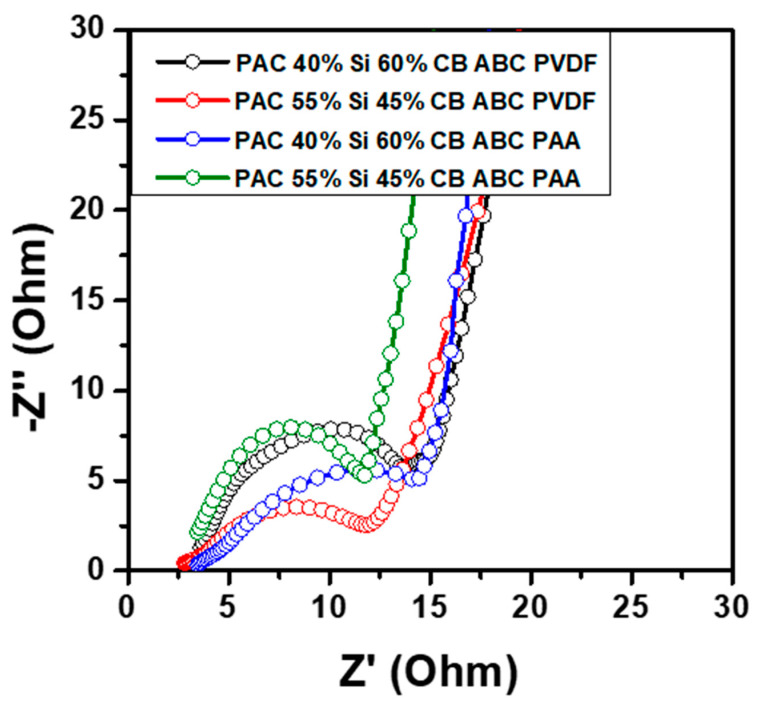
Nyquist plots from EIS measurement of PAC-Si-CB-ABC electrodes with different binders.

Figure 10 shows the EIS data for the PAC(40%)-Si(60%)-CB-ABC and PAC(55%)-Si(45%)-CB-ABC electrodes employing PVDF and PAA binders. The electrodes with PAC ratios of 40:60 and 55:45 and the PAA binder exhibited lower resistance values than those with PAC ratios of 40:60 and 55:45 and the PVDF binder. For both the PVDF and PAA binder systems, the impedance spectra displayed a semicircle in the high-frequency region, corresponding to the charge transfer resistance at the electrode–electrolyte interface, followed by a Warburg impedance in the low-frequency region, indicative of lithium-ion diffusion. The smaller diameter of the semicircle for PAC(40%)-Si(60%)-CB-ABC and PAC(55%)-Si(45%)-CB-ABC with the PAA binder indicates a lower *R_ct_*, with respective values of 14.8 and 15.5 Ω, compared to those of PAC(40%)-Si(60%)-CB-ABC and PAC(55%)-Si(45%)-CB-ABC with the PVDF binder (19.2 and 20.5 Ω, respectively); the data for the former indicate better conductivity and faster electrochemical kinetics. The reduced resistance at these ratios suggests more efficient ion transport and the formation of a stable SEI layer, which are critical for maintaining high performance during charge–discharge cycling [36,37]. The stability of these compositions was further confirmed by the linearity observed in the Warburg region, suggesting that the diffusion of Li ions within the electrodes was more uniform and less impeded, contributing to the overall stability and longevity of the battery performance. The results of this study align with those of previous research that emphasized the significance of the electrode composition and binder selection in enhancing battery performance. By carefully tuning the material ratios and employing effective binders, the internal resistance can be minimized, thereby improving the energy efficiency and cycling stability of lithium-ion batteries.

**Figure 11:**
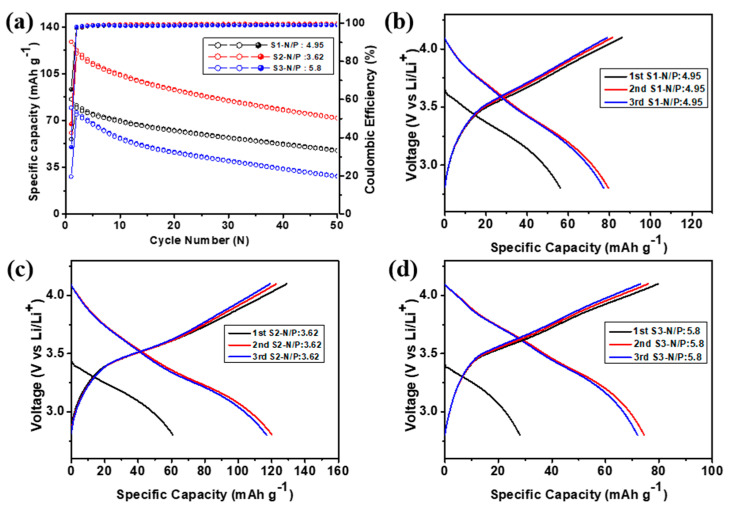
(**a**) Cycling performance, (**b**–**d**) initial charge–discharge profiles of the PAC(55%)-Si(45%)-CB-ABC||NCM622 full cells with different N/P ratios at 50 mA g^−1^.

As viable anode material, a full-cell test was performed with the NCM622 cathode. In the PAC(55%)–Si(45%)-CB-ABC||NCM622 full cells, the mass ratio of the anode/cathode was set at thiree different levels: 1:4.95 (S1), 1:3.6 (S2), and 1:5.8 (S3) as shown in Figure 11a. The electrodes were pre-lithiated by pre-cycling in a half-cell configuration prior to full-cell assembly. The full cell was operated at a current rate of 50 mA g^−1^ within the voltage window of 2.8–4.1 V. The charge capacity in the first cycle, calculated using the active mass of the cathode, was 85.98, 128.9, and 79.7 mAh g^−1^ for the S1, S2, and S3 full cells, respectively (Table 1 and in Figure 11c,d). In the second cycle, the coulombic efficiency improved significantly to 97.6, 97.9, and 98.0 % for S1, S2, and S3, respectively; thus, all full cells offer highly reversible electrochemical reactions with delivery of >98.5% CE from the 3rd cycle. The full cell capacity delivered after 50 cycles was 74.8, 72.0, and 28.5 mAh g^−1^ for full cells S1, S2, and S3, respectively, which indicates that the full cell with the 3.62 N/P ratio delivered 60% capacity retention. The average energy density was 217.8, 323.3, and 165.3 Wh kg^−1^ after the 50th cycle for S1, S2, and S3, respectively. In Figure 11, a comparison of the performance of the batteries employing the electrodes with different ratios indicated that S2 exhibited the highest initial charge capacity and relatively high capacity retention after cycling, as well as a higher energy density [38]. This indicates that the full cell with the S2 ratio is superior not only in terms of the initial charge capacity but also in terms of the long-term cycling performance [39].

## 4. Conclusions

Using high-energy ball milling (HEBM), advanced composites consisting of nanosilicon and porous artificial carbon, provided by Black Materials Co., Ltd., were successfully synthesized, resulting in an active material with uniformly distributed silicon within the PAC matrix. The addition of ABC significantly increases the surface area within PAC, thus expanding the pathways for lithium-ion movement, enabling stable charge–discharge cycling, and facilitating the development of high-capacity electrodes.

XRD, TEM, and EDS analyses confirmed the porous structure of the synthesized anode material. BET measurements indicated that the incorporation of ABC increased both the pore volume and specific surface area, providing more space for lithium-ion diffusion. Voltage profile analysis allowed for a detailed evaluation of the charge–discharge characteristics of the composite anode, revealing distinct peaks and stable cycling performance up to 100 cycles. GITT analysis demonstrated better ionic conductivity in the case of PAC(40%)-Si(60%)-CB-ABC and PAC(55%)-Si(45%)-CB-ABC with the PAA binder compared to that of conventional graphite, PAC(40%)-Si(60%)-CB-ABC, and PAC(55%)-Si(45%)-CB-ABC with the PVDF binder, thus demonstrating the excellent lithium-ion mobility of the composite anode material. This better performance may be due to the tight binding between Si and PAA, in addition to the use of CB as the conductive medium, as well as the porous sites resulting from ABC evaporation. Overall, the developed PAC(55%)-Si(45%)-CB-ABC with PAA binder demonstrated a reversible capacity of 850 mAh g^−1^ at 100 mA g^−1^ and a high-rate capability of 600 mAh g^−1^ at 2000 mA g^−1^. The full cell with the NCM622 cathode exhibited stable cyclability with a reasonable energy density of 323.3 Wh kg^−1^. The successful synthesis of such composite materials with PAC and ABC suggests promising avenues for future research aimed at developing high-energy-density full cells. By creating new active materials, the superior performance of nanosilicon and PAC composites can be harnessed, which will significantly benefit the battery industry.

## Figures and Tables

**Table 1:** Comparison of the electrochemical cycling performance of full cells with different N/P ratios.

Sample	1st Cycle Charge Capacity	CE (%) After 1st	CE (%) After 2nd	Working Voltage of Full Charge at 2nd Cycle	Capacity at 2nd Cycle (mAh g^−1^)	Capacity at 50th Cycle (mAh g^−1^)	% Retention After 50th	N/P Ratio	Average Capacity (mAh g^−1^) and Energy Density at 50th Cycle (Wh kg^−1^)
S1	85.98	65.3	97.6	3.587	81.4	48.3	59.3	4.95	61.19 (217.84)
S2	128.9	47.2	97.9	3.52	122.7	72.7	59.3	3.62	91.25 (323.3)
S3	79.7	35.6	98.0	3.62	75.9	28.5	37.6	5.8	45.66 (165.29)

## Data Availability

All materials, data, and associated protocols contained in this manuscript can be made available to readers upon request.

## Supplementary Materials

The following supporting information can be downloaded at: https://www.mdpi.com/article/10.3390/nano14231953/s1, Figure S1: The cycling property of Si-CB-ABC electrode.
