# Supplementary material for: Artificial Graphite-Based Silicon Composite Anodes for Lithium-Ion Batteries
Source: Nanomaterials (Basel). 2024 Dec 5;14(23):1953. doi: 10.3390/nano14231953 (PMC11643770; doi:10.3390/nano14231953)
Supplement: Supplementary file 1 [file nanomaterials-14-01953-s001.zip › nanomaterials-3341490-supplementary.pdf]

Supplementary Information

Artificial graphite-based silicon composite anodes for lithium-ion batteries

Sae Min Park[1,+], Tejaswi Tanaji Salunkhe[1,+], Ji Hyeon Yoo[1], Il Ho Kim[2], Il Tae Kim[1,*]

[1]Department of Chemical, Biological and Battery Engineering, Gachon University, Seongnam-si, Gyeong-gi-do, 13120, Republic of Korea

[2]Advanced Carbon Materials Center, Black Materials Co., LTD., Hwaseong-si, Gyeonggi-do, 18255

[*]E-mail address: itkim@gachon.ac.kr; Tel.: +82-31-750-8835 (I.T. Kim)

[+]They are equally contributed.

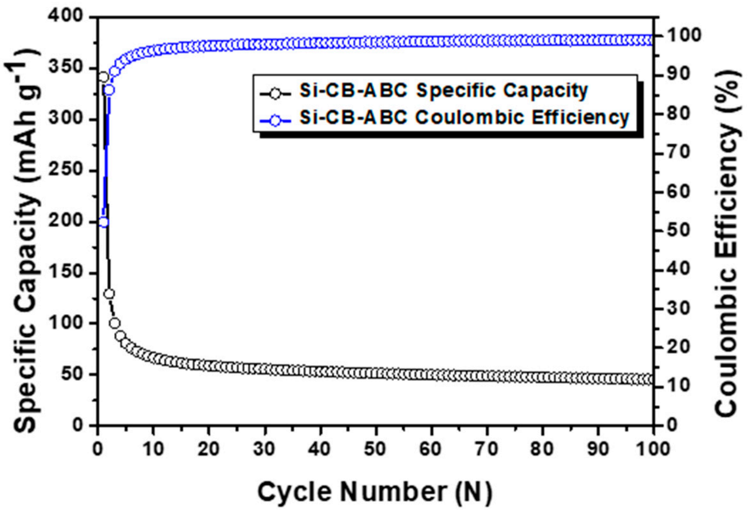

Figure S1. The cycling property of Si-CB-ABC electrode.
